# Predictive fetal medicine and the ownership of prenatal data: legal, ethical, and professional challenges

**DOI:** 10.3389/fdgth.2026.1758249

**Published:** 2026-01-21

**Authors:** Yoann Marechal

**Affiliations:** Pediatric and Neonatal Intensive Care, Hôpital Civil de Charleroi, Charleroi, Belgium

**Keywords:** antenatal exposure, fetal data, ownership (not absolute), predictive medicine, professional secret

## Abstract

Advances in artificial intelligence and multi-omic analysis are transforming fetal medicine from a diagnostic discipline into a predictive one. Yet the legal, deontological, and ethical frameworks that govern prenatal and fetal data have not evolved accordingly. Current regulations protect the mother as a patient but do not recognize the fetus—or the future child—as a legal data subject. As a result, information generated before birth remains confined within maternal medical records, creating uncertainty about who may later access or reuse it. This paper examines the emerging ethical and legal challenges of predictive fetal medicine, focusing on the transition from maternal consent to the child's future right to their own prenatal data. Through the lens of professional deontology and comparative law, we analyze the tensions between confidentiality, autonomy, and beneficence. We propose a framework of prenatal data stewardship, shifting from static notions of data ownership to shared responsibility across time. Establishing national or international repositories under transparent governance could enable ethical reuse of fetal data while safeguarding maternal privacy and ensuring future individuals' rights. Ultimately, aligning predictive fetal medicine with ethical and legal coherence requires collective action among clinicians, ethicists, jurists, policymakers, and industry. Only through such stewardship can information generated before birth become a trusted tool for care rather than control.

## 1 Introduction

Current prenatal and perinatal medicine primarily focuses on immediate outcomes: identifying malformations, guiding obstetric management, or supporting neonatal care. However, predictive medicine and big-data analytics are extending this horizon. Techniques such as the non-invasive prenatal test (NIPT), which analyzes cell-free fetal DNA in maternal blood, are now capable of capturing not only chromosomal anomalies but also epigenetic signatures that may reflect environmental exposures and predict later health risks (1, 2).

Recent research has suggested that fetal-origin molecular data may hold predictive value for long-term conditions, including metabolic disorders, neurodevelopmental variations, and cancer susceptibility (3, 4). Yet while these scientific capabilities expand, the legal and ethical frameworks surrounding data ownership, consent, and privacy have not evolved. The resulting uncertainty raises pressing questions: who owns fetal data, who may reuse them, and how should access evolve when the child becomes capable of decision-making?

The purpose of this paper is to clarify this emerging normative gap by reviewing the current legal, deontological, and ethical frameworks, and by proposing guiding principles for future governance of fetal data in predictive medicine.

## 2 Fictional case study

During a science project on environmental health, 11-year-old Lucas and his classmates learn about a new artificial intelligence (AI)-based “carcinogenic prenatal score”.

The algorithm, recently developed by an international consortium, analyses methylation patterns detected in cell-free fetal DNA (cffDNA) obtained during routine Non-Invasive Prenatal Testing (NIPT). Researchers claim that these epigenetic signatures can reveal whether an individual was exposed *in utero* to certain carcinogenic pesticides now banned in Europe and North America.

Lucas is intrigued. His mother died of breast cancer when he was three years old, and his father remembers that she had participated in one of the early NIPT research programs during pregnancy. When the father contacts the hospital to ask whether those prenatal data still exist, he learns that archived sequencing results from the study are indeed stored in a biobank, but that the material was collected under the mother's consent and therefore remains part of her medical record.

Lucas insists that the AI analysis could tell whether he is at risk. His father hesitates: would it be morally right—or perhaps even a parental duty—to request access to data that were never intended to belong to his son? The research team, for its part, faces a dilemma between scientific curiosity and respect for data protection law.

This fictional scenario illustrates the emerging tension between scientific potential, parental responsibility, and the absence of a clear legal subject for fetal data collected before birth. The example is intentionally hypothetical and reflects emerging lines of research rather than clinically validated tools currently available in routine care.

## 3 The legal and professional limbo of fetal data

### 3.1 The non-recognition of the fetus as a legal subject

Under existing legal frameworks, data-protection rights apply only to identifiable persons. The fetus, however, is not recognized as a legal subject. Within European Union law, including the General Data Protection Regulation (GDPR 2016/679), the data subject is defined as an identifiable natural person, presupposing juridical existence after birth (5). Consequently, fetal data—whether derived from ultrasound, MRI, amniocentesis, or NIPT—are part of the maternal record, governed by her consent and confidentiality obligations (6).

After birth, there is no automatic transfer of ownership or control to the child. Hospitals and research institutions therefore retain archived fetal datasets in a legal vacuum (1).

### 3.2 Consent, property, and secondary use

The GDPR regulates processing, not ownership. Consent given during pregnancy applies to a limited clinical purpose. When new uses—such as AI-driven reanalysis or predictive modeling—emerge decades later, the original consent no longer applies (1). The future child has no recognized right to claim or retract the maternal authorization under which data were collected. Some scholars have proposed treating fetal data as a continuing health asset that transitions to the child's control at birth (7), but no such mechanism currently exists in law.

### 3.3 Comparative perspectives

In the United States, sectoral laws like HIPAA (1996) and GINA (2008) provide limited protections but do not define ownership or postnatal access to prenatal data (8, 9).

In Japan, the Act on the Protection of Personal Information (APPI) and the Next-Generation Medical Infrastructure Law focus on anonymization and secondary use but remain silent on fetal datasets (10).

Globally, fetal data fall between categories: protected as part of maternal privacy, yet belonging biologically to another human being who is not yet a rights-bearing subject.

### 3.4 Professional deontology as a normative layer

Beyond law, professional codes provide moral and operational guidance. The World Medical Association's International Code of Medical Ethics (11), the Declaration of Geneva (12), and the Statement on Genetics and Medicine (13) emphasize confidentiality, autonomy, and primacy of patient welfare.

Applied to fetal data, these codes affirm that the pregnant woman remains the principal rights-bearing patient, and that her medical information—including fetal findings—remains confidential. Disclosure is permissible only under exceptional conditions of serious, preventable harm and minimal necessity (1, 13).

No deontological standard yet addresses long-term reuse of fetal-origin data for predictive purposes, leaving clinicians without clear ethical directives.

### 3.5 Parental representation and the evolving capacity of the child

After birth, parental guardians act on behalf of the child, but autonomy gradually increases with age. Some jurisdictions, such as Belgium, allow minors of discernment to make medical decisions independently, whereas others require parental consent until majority (6).

Ethically and legally, these asymmetries highlight the absence of a unified concept of evolving capacity within data protection law. A governance model that progressively transfers decision rights to the maturing child could reconcile autonomy with privacy while respecting the mother's original confidentiality.

### 3.6 Conclusion of the legal and professional analysis

Fetal data thus exist in a liminal space—collected for clinical care, yet potentially valuable for lifelong prediction. Neither law nor professional codes define their transition from maternal confidentiality to the child's autonomy. Predictive fetal medicine therefore operates in a normative vacuum where technology advances faster than the legal and professional capacity to regulate it.

In these circumstances, a child or future patient could face a “loss of opportunity” in the context of developing preventive medicine, should the absence of clear governance or data transition hinder access to timely interventions or personalized care.

## 4 Ethical challenges in predictive fetal medicine

### 4.1 Balancing beneficence and autonomy

Predictive fetal medicine seeks to prevent disease by acting early, even before birth. Yet such beneficence must respect autonomy—the rights of the mother and, later, of the child. The ethical tension lies in ensuring that data intended for protection do not become tools of surveillance or appropriation (1, 11).

### 4.2 Maternal autonomy and enduring confidentiality

Because fetal data originate from the pregnant body, they remain intertwined with maternal identity. Ethical governance must prevent retroactive data appropriation and maintain dual protection:

maternal data remain confidential unless separable from fetal data;
secondary use requires independent ethical oversight.

This preserves trust and respects deontological obligations of prudence and non-maleficence (11, 13).

### 4.3 The evolving capacity of the child

The principle of evolving capacity (14) supports progressive access: as the child matures, the child may claim rights over prenatal data concerning the child. However, disclosure must be proportionate and accompanied by counselling to prevent harm. Ethical governance must balance empowerment with protection. It must be acknowledged that access to prenatal predictive data is not ethically neutral. Reopening prenatal datasets may expose individuals to psychological distress, identity anxiety, or anticipatory stigma, particularly when probabilistic risk scores concern severe or late-onset conditions. There is also a risk of data misuse, including over-interpretation of uncertain predictions or secondary uses beyond the original clinical intent. These concerns underscore the necessity of proportional disclosure, counselling, and robust governance rather than unrestricted access.

### 4.4 Justice, transparency, and collective responsibility

AI-based predictive medicine risks reinforcing inequalities. Ethical frameworks such as the European Health Data Space (EHDS 2025) (7) and the UNESCO Recommendation on the Ethics of Artificial Intelligence (2021) (15) advocate transparency and fairness but must extend to prenatal data. Governance mechanisms should ensure that predictive tools serve equity, not exclusion.

### 4.5 From ownership to stewardship

Ethical reflection reveals three imperatives:

promote the child's best attainable health;
protect the autonomy and privacy of all individuals;
ensure fairness and accountability in data use.

Achieving this requires a shift from ownership to stewardship, where data are managed as a shared moral responsibility rather than private property. Predictive fetal medicine should thus become a model of care grounded in transparency, dignity, and trust.

## 5 Discussion and recommendations

The rapid expansion of predictive medicine and big-data analytics has profoundly transformed how health information is generated, stored, and interpreted. Yet the regulatory and professional frameworks governing prenatal and fetal data remain anchored in an era of episodic, physician-centred care. The legal concepts of patient, consent, and confidentiality—designed for discrete clinical acts—no longer fit a world in which health trajectories are continuously modelled from fetal life onward.

This misalignment is not merely procedural but structural: the law protects data of subjects who already exist, while predictive medicine increasingly concerns future persons. Professional deontology, grounded in individual relationships of trust, struggles to accommodate algorithmic ecosystems in which data circulate across decades, borders, and institutions. As a result, innovation progresses within a normative vacuum where the technical capacity to predict outpaces the moral and legal capacity to govern.

To address this gap, a new model of prenatal data stewardship is needed—one that unites the ethical principles of autonomy, beneficence, and justice with the technical realities of data-driven medicine. Such a framework could rely on three complementary pillars:

National or international repositories of fetal and perinatal data, established under strict governance and transparency standards. These repositories would integrate clinical, genomic, and imaging data while ensuring separation between maternal and fetal identifiers. Access would remain individual, avoiding secondary familial use unless explicitly authorized under ethical oversight.
Deferred ownership and consent mechanisms, allowing the child—once capable of discernment—to claim or manage access to their fetal data, with appropriate counselling and safeguards.
Ethical and public oversight bodies responsible for balancing individual rights and societal benefit, ensuring equitable access and preventing commercial or discriminatory misuse.

In practice, such a stewardship model could be operationalized through predefined governance checkpoints: initial maternal consent specifying potential future recontact; secure archiving of fetal-origin data under independent custodianship; and a deferred access mechanism enabling re-evaluation when the child reaches sufficient maturity. Multidisciplinary oversight committees—combining clinical, ethical, and legal expertise—could arbitrate access requests on a case-by-case basis, ensuring proportionality and psychological support.

By embedding these repositories within existing digital-health infrastructures—such as the forthcoming European Health Data Space (EHDS 2025) or interoperable global registries—societies could preserve the scientific value of fetal data while respecting evolving rights. This model would transform fetal information from a static medical record into a dynamic health resource, governed by principles of transparency, proportionality, and shared responsibility.

Ultimately, the ethical legitimacy of predictive fetal medicine will depend on our ability to design governance systems that evolve as knowledge does. Data generated before birth should not vanish in archives nor be confined by outdated legal categories; they should form part of a life-long informational continuum, accessible under conditions that respect both the privacy of origins and the promise of preventive care.

## 6 Conclusion and future perspectives

The future of predictive fetal medicine depends on stewardship rather than ownership. Fetal data should be governed as a shared societal resource—ethical, transparent, and reclaimable by those it concerns. Bridging the gap between technological progress and current law requires collective action from clinicians, ethicists, jurists, policymakers, and industry, ensuring that what begins as data before birth becomes a tool for equitable care, not control.

## Data Availability

The original contributions presented in the study are included in the article/Supplementary Material, further inquiries can be directed to the corresponding author.
